# A Community of Strangers: The Dis-Embedding of Social Ties

**DOI:** 10.1371/journal.pone.0067388

**Published:** 2013-07-04

**Authors:** Paolo Parigi, Bogdan State, Diana Dakhlallah, Rense Corten, Karen Cook

**Affiliations:** 1 Sociology Department, Stanford University, Stanford, California, United States of America; 2 Sociology Department, Tilburg University, Tilburg, The Netherlands; University of Warwick, United Kingdom

## Abstract

In this paper we explore two contrasting perspectives on individuals' participation in associations. On the one hand, some have considered participation the byproduct of pre-existing friendship ties — the more friends one already has in the association, the more likely he or she is to participate. On the other hand, some have considered participation to be driven by the association's capacity to form new identities — the more new friends one meets in the association, the more likely he or she is to participate. We use detailed temporal data from an online association to adjudicate between these two mechanisms and explore their interplay. Our results show a significant impact of *new* friendship ties on participation, compared to a negligible impact of *pre-existing friends*, defined here as ties to other members formed outside of the organization's context. We relate this finding to the sociological literature on participation and we explore its implications in the discussion.

## Introduction

Associations between individuals are considered fundamental to the lives of communities and significant for the proper functioning of democracies [1]–[4]. An active associational life has also been linked to the emergence of collective action and to an increased capacity for resolving social dilemmas [5], [6]. However, the determinants of individual participation in associations are not fully understood [7], [8]. Two competing explanations of participation are proposed: (a) participation is the byproduct of having multiple pre-existing friendship ties with individuals in the association [9], [10] or, (b) participation is the result of the association's capacity to promote new identities among its members [11]. In the first view, the associational life of an individual benefits from embedding involvement in the association in her own social networks; for example, by having her friends join the association she is a member of or by simultaneously joining the same association with her friends. In the second view, associational life benefits from the association promoting a process that pushes in the opposite direction, i.e., toward dis-embedding engagement with the association from a member's pre-existing social networks; for example, by promoting ties to new friends through the association.

The difficulty in adjudicating between these two mechanisms resides not just in the fact that they are clearly interconnected, but also in the existing lack of detailed temporal data for determining the causal relationships between them and their varied effects on associational engagement. In the last decade or more, the Internet has emerged as a significant site for the promotion of associational life. An increasing number of associations have originated online and have become significant producers of meaning in the daily lives of their members. Associations born on the Internet typically collect temporal data on their members' social networks and these data have captured the attention of a number of social scientists [12]. We use social network data collected by one of these associations to determine the relative contribution of the proposed mechanisms to individuals' levels of participation.

Our results suggest that having many prior friends in the association *decreases* participation, while forming new friends through the association *increases* participation. We relate this finding to the sociological literature on the role of networks in the mobilization of individuals. In particular, we adopt Gould's argument that mobilization occurs along a continuum bounded on the one hand by established solidarities and on the other hand by new identities [7]. The role of pre-existing networks in explaining participation shifts from strong to weak along the continuum between these two poles. Our findings suggest that online associations may benefit more from promoting a new identity for their members rather than from embedding the association into the pre-existing social networks of their members. We discuss the main implications of this argument in the conclusion.

## Following Friends or Building New Identity

Writing in the 1960s, Almond and Verba argued that what made Western democracies effective was active citizen participation in civic affairs. They developed what was called a “psycho-cultural approach to the study of political phenomena” [1]. According to this perspective, individual values and beliefs explain why some societies appeared more vibrant than others in creating associations and, consequently, why democracies differed in efficiency despite sharing similar institutions like universal suffrage, division of power, constitutions, free elections, and so forth. Similarly and more recently, the work of Robert Putnam [4] on Italian regional and local governments argued that regions in Northern Italy were better governed because of a longer tradition of civic associations compared to the regions in Southern Italy. Putnam explains the relationship between strong networks of citizen participation and positive institutional performance in terms of ``social capital'' — the informal networks, norms of reciprocity and trust that are fostered among members of the same association.

While the two studies cited above stress the importance of macro level values and belief systems, several social scientists have articulated a structural perspective to explain the importance of associations and collective action more broadly [5], [13]. From this perspective, social networks have emerged as a key factor in understanding modern associations [14]. Wellman, for instance, argued that contemporary associations and communities are better conceptualized as networks that are either locally or globally bounded rather than densely-knit, village-like groups [15]. Yet, within the literature that focuses on associations as networks tension, has emerged between two approaches. On the one hand, scholars have highlighted the role of social networks, and of friendship in particular, for explaining why people participate in associations. On the other hand, scholars have stressed the importance of identity processes in explaining the growth of associations. People join because the association provides them with a new identity and new circles of friends that they are interested in acquiring. With some notable exceptions, both approaches draw heavily from research on social movements.

The classic view of the importance of pre-existing network ties is perhaps articulated best in McAdam's *Freedom Summer* [16]. McAdam shows that activists who participated in the 1964 Mississippi summer camp were more likely to have friends already at the camp. McAdam and Fernandez analyzed the role of networks by studying the centrality of the people recruited to participate in the summer camp [17], further confirming the importance of pre-existing ties. The importance of network ties for participation received formal treatment by Centola and Macy [10]. Using a simulation model, Centola and Macy studied the diffusion of collective action with respect to different levels of risk. Innovation in mobilization tactics cascaded through the network when risk was minimal and thus adoption thresholds were low. The diffusion process stopped at local bridges, however, when the required threshold of adoption, i.e., the number of prior friends that have adopted, increased. In sum, this literature suggests that network ties matter for participation particularly when the risk involved in participating is perceived to be high.

In *Peasants into Frenchmen* [18], Eugene Weber articulated, on historical and institutional grounds, a view counter to the importance of pre-existing friendship ties for explaining participation. Weber describes the ways in which the French state facilitated the creation of a national identity among the locally identified members of the population by enabling contact among the soon-to-become ``French'' individuals from the country's provinces. National universities, military service, corporations, and administrative bodies all facilitate the meeting of people from various parts of the state's territory. With contact comes the opportunity for the development of social relations, and the formation of such relations confirms one's loyalty to the nation. Membership in these institutions promotes new identities, which in turn influence participation.

While Weber's argument focuses on institutions rather than associations, a similar argument about the importance of identity has been advanced by another set of social movement scholars interested in processes of identity formation and collective action [13], [19]. Deborah Minkoff, for instance, argued that those in certain disadvantaged categories such as gays and lesbians, the elderly, and women, lacked access to the infrastructure that facilitates generation of ties between members. Mobilization of these groups created identities that then produced social ties [20]. Further reinforcing this argument and providing a more formalized approach to it, Kairam and co-authors used data from Ning online communities to show the existence of a non-network growth model for communities and, by extension, for associations [11]. In this case individuals join because they share a common interest with the community. The new relationships individuals form with members of the association subsequentily helped to promote a new identity.

To a certain extent the two mechanisms promoting participation are interrelated. Pre-existing friendship ties may be the first reason why one decides to begin to participate, but the capacity to form new friendships within the association may be the reason why one decides to continue participating. Roger Gould explored this interplay of forces directly in his analysis of the Paris Commune uprising in 1870. He argued that when mobilization occurs along the lines of pre-existing networks (e.g. the National Guard enlistment in Paris mirrored neighborhood boundaries) protest is more efficient but fails to produce extra levels of commitment to the organization or association beyond those already created by neighborhood ties[7]. In this case, the association does not have an independent identity. Conversely, when the association cuts across pre-existing networks, mobilization is harder to achieve unless it manages to create a total institution. In such a scenario, the association develops a strong identity of its own. In line with this reasoning, in a different case study, Peter Bearman shows that Confederate army battalions that had soldiers enlisted from the same region had more desertions during the Civil War than more regionally heterogeneous battalions (everything else being equal) [21]. The embedding of ties in pre-existing solidarities weakened the independence of the organization, in this case the Army battalion, leading to the quicker demise of mobilization during the final months of the war.

In light of this research we represent the role of pre-existing networks in participation along a continuum: at one end of the continuum, pre-existing ties are important and the association has a weak identity; at the other end, pre-existing networks are not relevant and the association has a strong identity.

## Associations, Social Networks and the Internet

The question of whether the rise of the Internet has positive or negative effects on associational life and community formation has been the topic of heated debate. Early on, a ``dystopian'' line of scholars feared that the use of Internet would erode communities and social relations. Internet use, they argued, would replace in-person interaction and long-distance online interaction would replace social interactions in local communities [22]. At the same time, a ``utopian'' line of thought predicted that the Internet would enhance, or even radically transform, traditional forms of social capital [15]. Indeed, some early studies suggested that online interaction via e-mail and online discussion boards could strengthen local communities [9].

In the meantime, the nature of online interaction has evolved rapidly, most notably through the surprisingly swift rise of online social networks such as Facebook, MySpace and Twitter. While older forms of online social interaction such as e-mail seemed especially suited to support existing offline interaction structures, these newer online social networks allowed users not only to interact with their own contacts, but also to traverse the network by discovering the contacts of their own contacts [23], thereby making offline social structure potentially less relevant. Online social networks seem especially suited for the creation of new connections that bridge social contexts and thus may have an ameliorating effect on the cleavages found in modern societies, cleavages typically reinforced through certain patterns of offline interactions. Recent empirical research [24] suggests, for example, that while online networks are firmly rooted in existing offline social networks, they are positively associated with various forms of bridging social capital.

In a parallel debate, social movement scholars have emphasized the increasing importance of information and communication technology (ICT), and the Internet in particular, for social movements, arguing that social movements rely on networks to provide organizational structure and that ICT facilitates such networks [25]. Although much research on the relationship between social movements and online interaction is based on studies of older types of online interaction (such as e-mail, in contrast to modern online social media such as Facebook and Twitter), this literature initially predicted that ICT would be most valuable to social movements that are either large, well-established and centrally organized, or social movements that consist of informal and geographically dispersed networks [25]. These predictions seem largely at odds with recent developments such as the ``Arab Spring,'' in which online social media (i.e., Facebook) appear to have been pivotal in coordinating local social movements [26]. In sum, scholarly work on the role of the Internet and ICT in contemporary societies has highlighted, on the one hand, how a type of social capital can be created in online interactions and, on the other hand, how ICT can be used to create global and local mobilization.

Regardless of perspective, whether the Internet promotes more participation or less and whether ICT favors global or local collective actions, associations that have their origins online have gone on to become significant producers of meaning in the daily lives of their members. We use social network data collected by one of these associations to determine the impact of the two distinct mechanisms identified above on individual levels of participation. The theoretical question we explore is thus: *Do online associations primarily generate participation by tapping into pre-existing networks of their members or by promoting a new identity independent of these networks*?

## Data and Methods

For our analysis we use a unique dataset collected by CouchSurfing.org–the world's largest and fasted growing social travel network–whose stated mission is to “to make the world a smaller and friendlier place, one life-changing experience at a time.”' After negotiating access to the data with CouchSurfing and signing a Non-Disclosure Agreement with them, we submitted our research protocol to the Internal Review Board (IRB) of Stanford University that determined that it posed no more than minimal risk (``Notice of Exempt Review,'' received in April 2012). All the data we used in our analysis were anonymized. Members of CouchSurfing engage not only in hospitality exchanges, visiting each other often in disparate corners of the world, but also in organizing interest groups and social gatherings for members that live in the same area. Local chapters of CouchSurfing resemble leisure associations whose goal is to facilitate interactions among their members. While some interactions occur offline, it is by logging into their personal webpages that members learn about gatherings and about the local initiatives of the various interest groups. In other words, on CouchSurfing the online platform plays a key role in facilitating the offline meeting of like-minded people. For this reason, we use logins as an indicator of the overall level of individual participation with the life of the association.

Compared to traditional social networking websites, CouchSurfing's online platform introduced a number of innovations that arguably resulted in more credible data being collected about social ties. Rather than simply allowing users to add ``friends'' or ``buddies,'' CouchSurfing's platform asks probing questions about new social connections – whether the tie is one's best friend, a good friend, or a mere acquaintance, how much trust the friends have in each other, as well as the context in which the two individuals met. Even among the numerous online platforms that collect social network data, few gather network data at this level of detail. As a consequence, CouchSurfing has attracted a good deal of attention from the scientific community and from mainstream media [27]–[29]. We contend that data collected through such a strategy are likely to reflect meaningful social ties.

**Figure 1:**
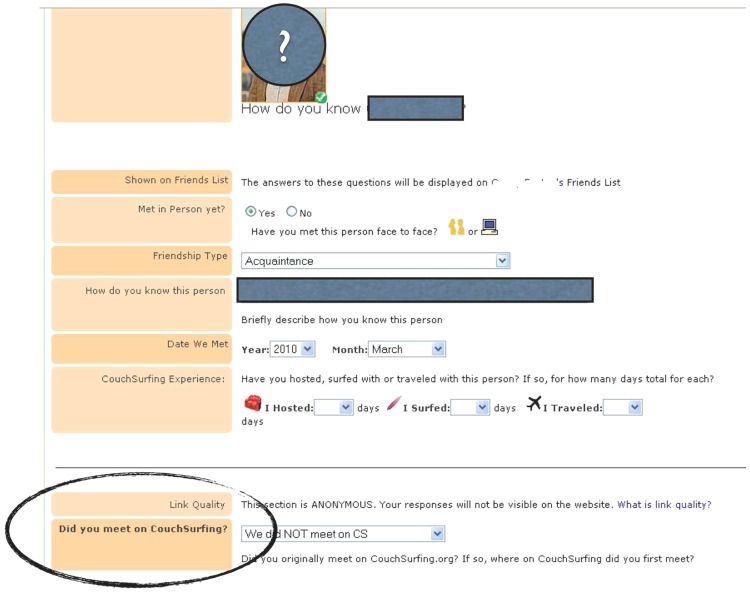
The information members enter after a friendship tie is reported on the website.

The period of our analysis spans the time from September 2003 to December 2010. We created a random sample of 10,000 American users, whose monthly logins were counted and recorded over their career in the association. Among American users, 43% are females, 75% are 29 years old or younger and, on average, have hosted or surfed, i.e., visited somebody, for 4.38 days and 4.51 days respectively. Figure 1 shows the information recorded by CouchSurfing for each tie a member reports with another member. We made the page completely anonymous by eliminating the names and pictures of all tie recipients. We used the information from the circled question (bottom left corner) to capture the extent to which CouchSurfing facilitated the formation of a friendship tie. In the example, the focal member reported that the tie predated membership in CouchSurfing. We called this type of relationship an *external tie*; we labeled a tie formed because of CouchSurfing an *associational tie*. Thus and within the scope of our argument, external ties capture pre-existing friendships formed outside of the organization while associational ties capture new friendships formed within the organization. Of the ten thousand members in our random sample, 2,941 of them acquired either or both of these types of ties during the period of analysis.

These data allowed us to test the impact of pre-existing relationships, i.e., external ties, on participation (logins) controlling for the individual propensity to form new friendships within the association (associational ties). The model that we used for our estimates, a panel vector autocorrelation model (PVAR), took into account the possibility of feedback loops among all the variables in the model[30]. In particular, the PVAR model controlled for the possibility that having many pre-existing friends joining the association reinforced the likelihood of forming new ties, that is, of developing a new identity. More formally the model is specified as:
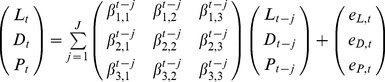
(1)


The variables in the model are 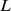, 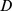 and 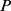 that respectively represent logins, nodal degrees (total number of friends, however acquired) and proportion of associational ties at time t. Each variable is time-demeaned to take into account any secular trends. We controlled for heteroskedasticity by dividing each variable by its time dependent standard deviation. We addressed autocorrelation of the individual observations by subtracting the forward mean, which corresponds to the mean of all future observations for each individual (Helmert transformation). Time was measured in month intervals.

In the formula above, betas represent the estimated coefficients of the effects of each variable on the other variables in the model and 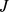 is the order of the model. The Lagrange multiplier test indicated that using three-month lagged variables did not create serial correlation. Furthermore, using a lag of three months maximized the Akaike Information Criterion (AIC) and improved the fit of the model. The estimates were obtained using the software Stata and the panel VAR procedure developed by Love and Zicchino [30].

We also considered how the PVAR system reacted to external shocks (Impulse-Response Function, or IRF). Unexpected shocks are defined as the impact of a one standard deviation increase of the error terms (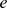) on the variables in the system. Since the PVAR model does not differentiate between endogenous and exogenous factors, analyzing the reaction of logins to a shock in one of the other variables estimated in the model allowed us to isolate the explanatory role of nodal degree (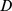) and proportion of associational ties (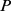) on participation.

We focused in particular on the role of associational ties, i.e., on the impact of a shock in 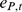 on logins 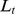 for 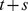 time periods. We set s to be equal to five so that the IRF estimated the impact of the shock for a six month period. We used Monte Carlo simulation with 500 repetitions to generate 95% error bands. This made possible estimation of the causal role of associational ties on participation within the constraints of the model.

## Results

**Figure 2:**
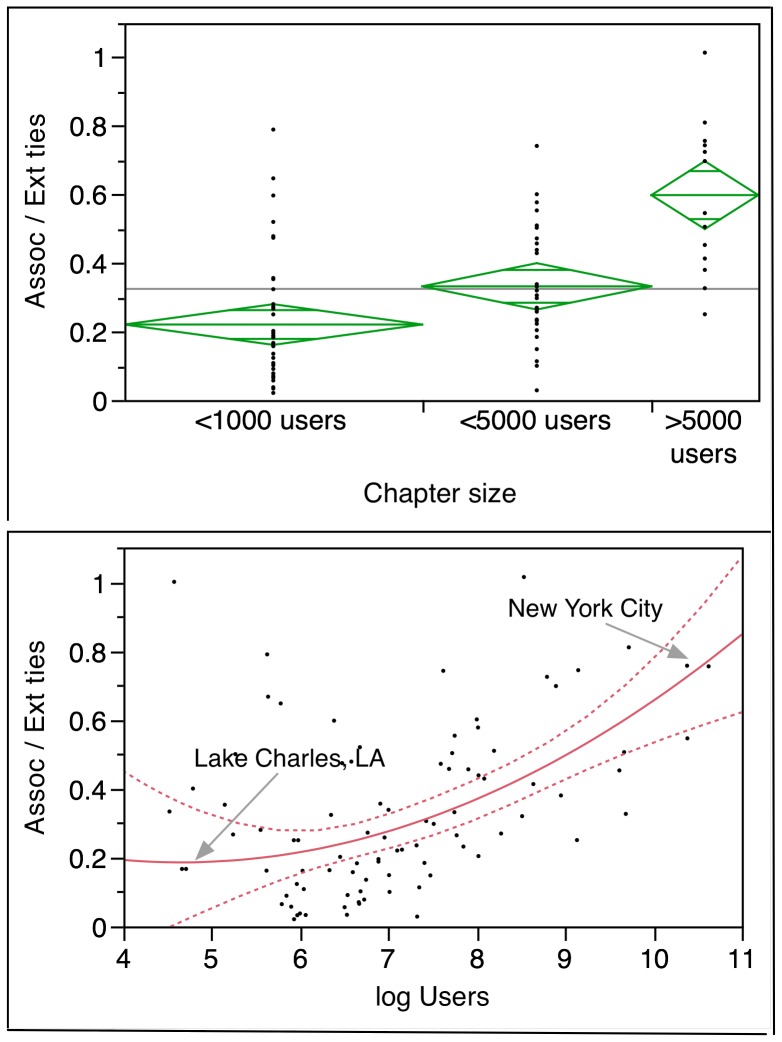
Ratio of ties by size of local chapters (top panel) and by (log) number of members (bottom panel). Each dot represents the total proportion of the two types of ties in a metropolitan area of the United States (CSA) as of December 2010 (N = 83.). We excluded CSAs where more than 90% of the members had no ties. In the top panel diamonds show the group means with 95% confidence intervals (One Way ANOVA, F ratio = 21.22, 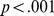). The bottom panel shows a quadratic fit to the data with 95% confidence interval. 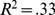.

Before estimating the PVAR model we explore the possibility that the propensity to form new friendships and, consequently, of participating more in the life of the association is strongly influenced by the size of the local chapter. For each member in our sample, we identified the metropolitan area of his or her residency. We matched these areas with the combined statistical areas (CSAs) as defined by the U.S. Census Bureau. Figure 2 (top panel) suggests that the greater the size of the local chapter, the more new social ties members acquire through CouchSurfing. The mean ratio between the two types of ties for small chapters (less than a 1,000 members) is.22 while for larger chapters (more than 5,000 members) is almost.60. Yet, a closer examination reveals that the relationship between the type of ties and the size of the chapter is rather weak. The bottom panel of Figure 2 shows the fit of a quadratic function, producing a 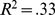. Not surprisingly, chapters in larger CSAs appear to have higher ratios than chapters in smaller CSAs. Thus, CouchSurfing members located in New York City had a greater proportion of associational ties compared to CouchSurfing members in Lake Charles, LA. More members create more opportunities for interactions among previously unknown members, making it easier for two strangers to meet.

**Figure 3:**
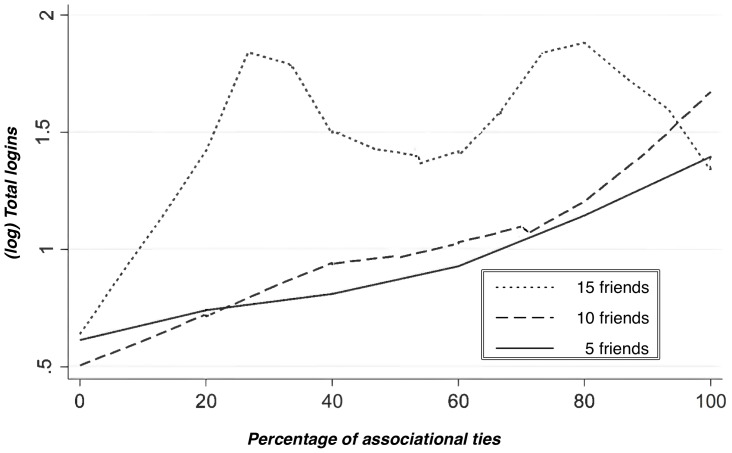
Total logins by number of associational ties.

While the aggregate analysis of the type of ties by the size of the chapters does not reveal a strong association between the two, we examine the extent to which opportunities to meet strangers within the association are structured by the overall number of friends one has (Figure 3). In the figure, people with 15 friends (top line) have a consistently higher proportion of associational ties than people with smaller circles of friends–5 and 10 friends–and, at the same time, participate more in the life of the association. Independent of the size of the local chapter therefore, the formation of new friendship ties, i.e., of associational ties, appears strongly impacted by the individual general propensity to form friendships. Figure 3 reinforces our confidence in focusing the analysis on individual level determinants of participation rather than at the aggregate level of local chapters.

**Figure 4:**
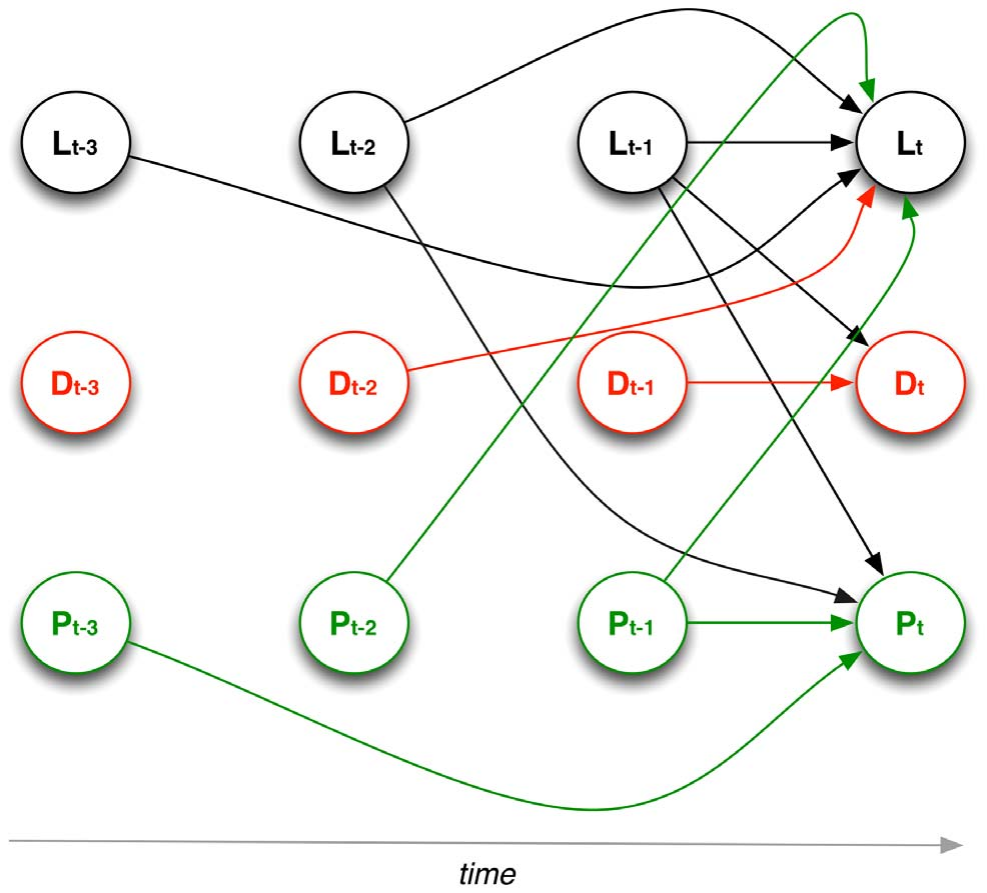
Graphical representation of the PVAR model with a three-lag period. L = number of logins (logs); D = number of ties; P = proportion of associational ties. Arrows represent statistically significant effects (at the 95% confidence level or more). Magnitude of the effects reported in Table 1.

Table 1 reports the 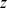-score estimates of the PVAR model while Figure 4 shows the graphical interpretation of the same model. We focus the discussion on the latter. Figure 4 suggests that while having many friends in the association has a two-month lagged impact on decreasing logins (negative coefficient in Table 1 for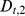 on 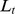), every increase in the proportion of friends met through CouchSurfing offsets this decrease in logins by almost one percentage point (a lagged effect of one month). After two months, associational ties lose their positive impact on engagement and behave more like external ties, i.e., they negatively affect logins. Yet, the magnitude of this latter effect is considerably smaller (see Table 1). Furthermore, the model indicates that the proportion of associational ties does not correlate with the total number of friends, and vice-versa. These findings suggest the existence of a separation between one's pre-existing networks of friends and the new circle of friends met via membership in the online association. Such a separation is strongly reinforced by one's participation in the association (compare the magnitudes of the effects of 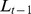 on 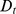 and 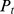).

**Table 1:** PVAR Estimates.

Dependent Variable	(Unlagged)		Ln (logins): L			Degree: D			% Assoc. Ties: P	
	Lag (months)	Coef.	(S.E.)	t-statistic	Coef.	(S.E.)	t-statistic	Coef.	(S.E.)	t-statistic
Ln (logins)	One	0.475***	(0.007)	69.659	0.095***	(0.011)	8.448	0.531***	(0.043)	12.282
	Two	0.075***	(0.006)	13.296	0.002	(0.007)	0.359	−0.066*	(0.033)	−2.028
	Three	0.055***	(0.005)	11.998	0.004	(0.006)	0.620	0.002	(0.025)	0.085
Degree	One	0.007	(0.015)	0.454	1.010***	(0.062)	16.231	−0.034	(0.036)	−0.946
	Two	−0.034**	(0.013)	−2.605	−0.094	(0.051)	−1.845	0.014	(0.036)	0.376
	Three	0.016	(0.008)	1.923	0.020	(0.034)	0.592	0.023	(0.024)	0.967
% Assoc. Ties	One	0.009***	(0.002)	6.295	−0.002	(0.002)	−1.320	0.878***	(0.010)	86.283
	Two	−0.003**	(0.001)	−2.670	−0.001	(0.002)	−0.398	−0.008	(0.007)	−1.051
	Three	0.000	(0.001)	0.533	−0.001	(0.001)	−0.724	−0.010*	(0.004)	−2.442

*Source*: CouchSurfing US dataset. Legend: *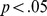, ** 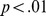, ***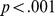. N = 67,183.

Each variable is time-demeaned to take into account any secular trends. We controlled for heteroskedasticity by dividing each variable by its time dependent standard deviation. We addressed autocorrelation of individual observations by subtracting the forward mean, which corresponds to the mean of all future observations for each individual (Helmert transformation). The reported coefficients are 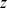-scores.

We also explore the impact on logins of a shock in associational ties and track this effect in time for six months. Such a shock rapidly increases logins in the first month, producing an increase of 4.3% in logins and remains largely constant at this level for the next five time periods. This suggests that the positive impact of a dis-embedding process on associational engagement is stronger at the beginning of one's membership in the association rather than later. Further, the consistency of the effect in time suggests that the dis-embedding process has a lasting impact on participation.

## Conclusions

Recently we have witnessed rapid growth in both associational life and the mobilization of collective action facilitated by the Internet. It is not hard to imagine a proximate future in which many human associations will be overwhelmingly made up of online communities. Individual social networks have been thought to produce positive effects on associational life by encouraging participation. Our analysis compares the effects of two competing mechanisms underlying individual levels of participation. We discovered that pre-existing social networks do not always promote associational life. In fact, while forming new social ties in the association does increase engagement, interestingly, having one's prior friends join the association you belong to actually decreases your overall engagement with the organization.

In light of sociological work on the role of pre-existing networks on the mobilization of collective action, our main finding suggests that CouchSurfing succeeded in forming a strong identity among its members, thereby reducing the importance of embedding participation within pre-existing networks to foster participation. This finding is also consistent with Centola and Macy's analysis [10] if we assume that joining a local CouchSurfing chapter is a low risk activity. Whether our finding is the byproduct of the nature of the specific association we targeted or is instead a characteristic of online associations, we cannot directly determine from our analysis. It is nevertheless useful to entertain the possibility that a potential effect of online associations would be that of promoting new identities that are independent of pre-existing ones.

Regardless of the precise scenario, we believe the study of online platforms for social networking such as the one provided by CouchSurfing can provide important insights into the world of all associations and the nature of their broader social, political and economic consequences. While the limited availability of data has restricted the scope of the debates about links between personal networks and levels of associational engagement in the case of primarily face-to-face associations, new data collection capabilities can give us important new insights into the complex nature of the interaction between networks and associational life. Further investigation is required to explore the similarities and potential differences between membership in online associations and those that are primarily face-to-face as well as the consequences of such membership for participation in civic activities.
